# Development of Certain Protein Kinase Inhibitors with the Components from Traditional Chinese Medicine

**DOI:** 10.3389/fphar.2016.00523

**Published:** 2017-01-09

**Authors:** Minghua Liu, Ge Zhao, Shousong Cao, Yangyang Zhang, Xiaofang Li, Xiukun Lin

**Affiliations:** Department of Pharmacology, School of Pharmacy, Southwest Medical UniversityLuzhou, China

**Keywords:** Traditional Chinese Medicine, protein kinase inhibitors, anticancer activity, PI3K/AKT/mTOR, MAPK, ERK

## Abstract

Traditional Chinese medicines (TCMs) have been used in China for more than two thousand years, and some of them have been confirmed to be effective in cancer treatment. Protein kinases play critical roles in control of cell growth, proliferation, migration, survival, and angiogenesis and mediate their biological effects through their catalytic activity. In recent years, numerous protein kinase inhibitors have been developed and are being used clinically. Anticancer TCMs represent a large class of bioactive substances, and some of them display anticancer activity via inhibiting protein kinases to affect the phosphoinositide 3-kinase, serine/threonine-specific protein kinases, pechanistic target of rapamycin (PI3K/AKT/mTOR), P38, mitogen-activated protein kinase (MAPK) and extracellular signal-regulated kinases (ERK) pathways. In the present article, we comprehensively reviewed several components isolated from anticancer TCMs that exhibited significantly inhibitory activity toward a range of protein kinases. These components, which belong to diverse structural classes, are reviewed herein, based upon the kinases that they inhibit. The prospects and problems in development of the anticancer TCMs are also discussed.

## Introduction

The protein kinase family encompasses all enzymes in the human body that catalyze the transfer of a phosphate group from a high energy molecule such as adenine triphosphate (ATP) to a specific amino acid in a protein. The human genome codes for more than 500 different protein kinases, which are divided into different families according to their selectivity for substrates (Sharma et al., 2008). Protein kinases play important roles in regulating cellular functions, including proliferation, survival, apoptosis, motility as well as metabolism and DNA damage repair, etc. Protein kinases such as cellular Src (c-Src), c-Abl, mitogen activated protein kinase (MAPK), phosphotidylinositol-3- kinase (PI3K), serine/threonine -specific protein kinase (AKT) and the epidermal growth factor receptor (EGFR), are commonly activated in cancer cells and known to play roles in tumorigenesis. Many of these occur in the same signaling pathway; EGFR kinase family members (HER1 [EGFR], HER2, HER3, and HER4) transmit signals through MAPK and PI3K to promote cell proliferation (Goldstein et al., 2008). The central role of kinases in virtually all networks of signal transduction is the driving motivation useful for the development of compounds modulating their activities (Ferré et al., 2014).

**Figure 1:**
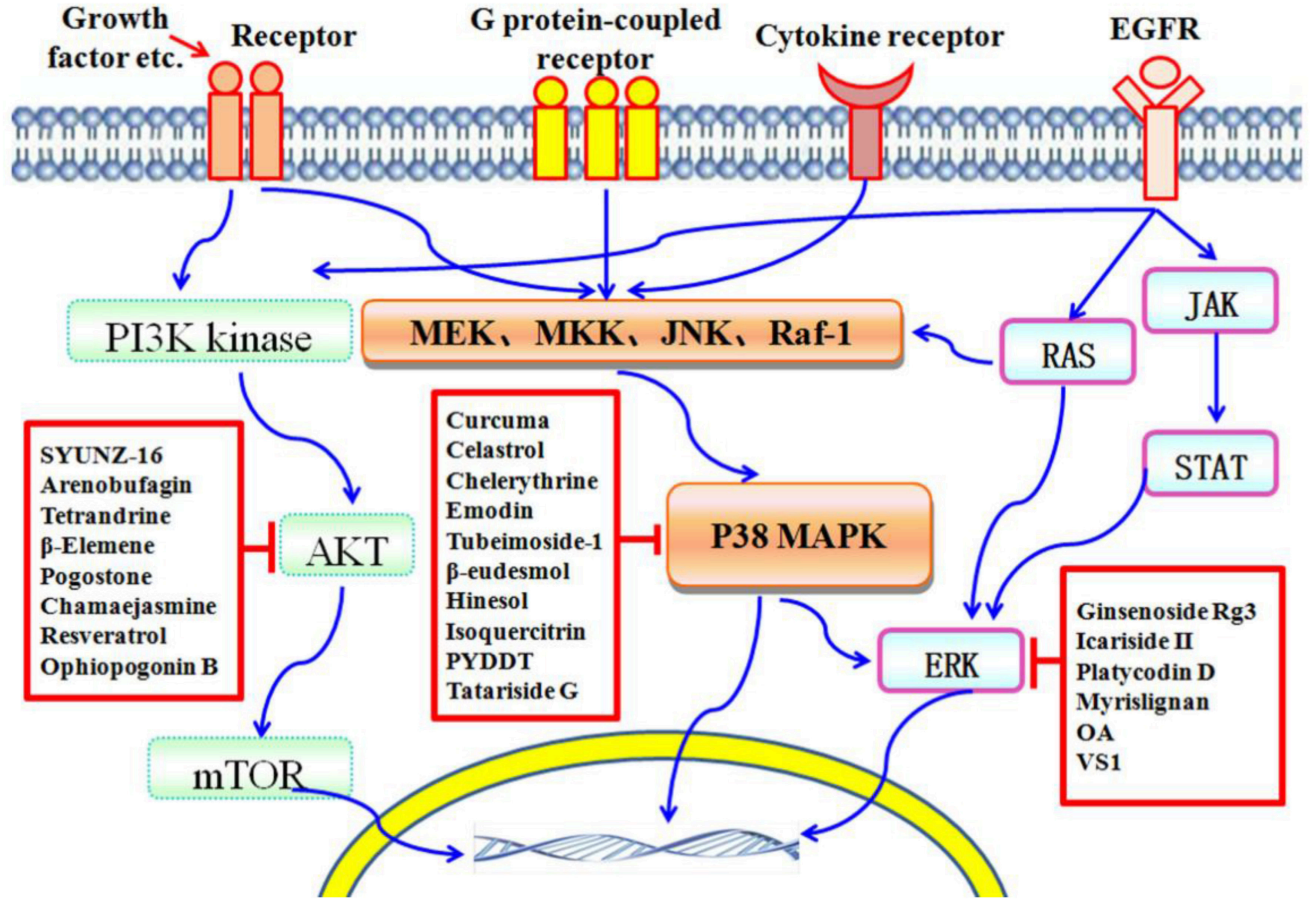
**Schematic depiction of TCM components that serve as protein kinase inhibitors**. The PI3K/AKT/mTOR, P38 MAPK, and ERK pathways are important players in cell death induced by protein kinases. Many compounds isolated from TCMs are capable of inducing cell death via affecting different protein kinase pathways.

In the past decades, numerous natural compounds with inhibitory effects on protein kinases have been identified or developed and some of them have been approved by the Federal Administration Agency (FDA) in the United States and used successfully in the treatment of human cancers clinically. In recent years, an increasing number of novel compounds have been isolated from Traditional Chinese Medicines (TCMs), and many of them have been reported to possess potent anticancer activity via inhibition of protein kinase mediated signaling pathways, including PI3K/AKT/mTOR, P38 MAPK, and ERK (Figure 1). In this mini-review, we focus on those compounds isolated from TCMs with inhibitory effects on protein kinases and present an overview of their anticancer effects and potentials in pharmaceutics for cancer therapy.

## Components isolated from TCMs that inhibit MAPK

MAPK, a serine/threonine specific protein kinase, regulates a variety of biological processes including cell survival, proliferation, differentiation, and apoptosis through downstream signal transduction cascades (Sun et al., 2010). The classical MAPK family consists of three subfamilies, i.e., the ERKs, the c-Jun N-terminal kinases (JNKs)/stress-activated protein kinases (SAPKs), and the p38 MAPKs (Chang and Karin, 2001; Kannan-Thulasiraman et al., 2006). JNKs and p38 MAPKs play critical roles in the signaling mechanisms that orchestrate cellular response to various types of cellular stress (Dhanasekaran and Reddy, 2008; Wagner and Nebreda, 2009). It has been acknowledged that the ERK signaling pathway is also very important in carcinogenesis. Selective inhibitors of these kinases are likely to affect cellular events with high specificities and are therefore the molecules of significant interest for discovery and development of anticancer pharmaceuticals (Nakao and Fusetani, 2007).

**Figure 2:**
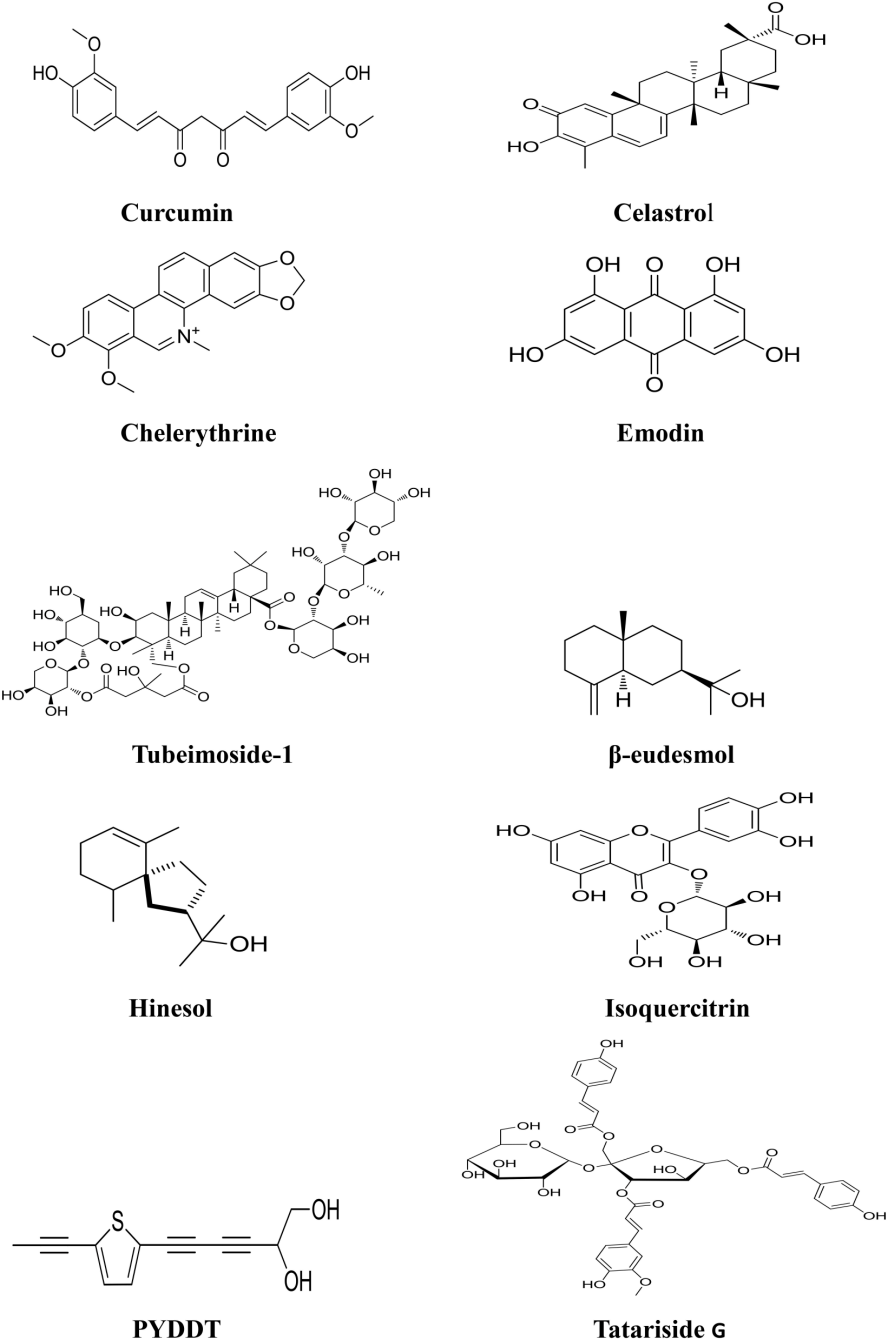
**Chemical structures of components isolated from TCMs with inhibitory effects via MAPK pathway**.

*Curcuma longa L*. is an important herb used in TCM to treat various types of pain and inflammation. Curcumin (Figure 2), an apolyphenolic compound, is isolated from the rhizomes of *Curcuma longa L*.. Recent studies have shown that curcumin has anti-tumor effect to inhibit cell proliferation and promote cell apoptosis in several types of cancer including hepatocellular carcinoma, lung cancer, breast cancer, colorectal cancer, etc. (Johnson et al., 2009; Banerjee et al., 2010; Cheng et al., 2010; Saha et al., 2010). Curcumin significantly activates the JNK and p38 MAPK, but not the ERK, signaling pathways via phosphorylation, thus down-regulating anti-apoptotic proteins Bcl-2, Bcl-XL, Mcl-1, and survivin in human HCT-116 colon cancer cells during apoptosis process (Collett and Campbell, 2004). Curcumin induces apoptosis of THP-1 human monocytic leukemia cells by activation of the JNK/ERK signaling pathway (Yang et al., 2012). Curcumin can also block cell cycle progression at the G_2_/M phase and induce apoptosis by regulation of ERK1/2 phosphorylation in nasopharyngeal carcinoma cells (Wang et al., 2013). Curcumin halts the growth of human HepG2 liver xenograft tumors in nude mice. Curcumin down-regulates the expression of p-ERK1/2 and p-AKT in tumor tissues by immunohistochemical analyses (Chintana et al., 2011). Furthermore, curcumin inhibits proliferation of colorectal carcinoma cells by modulating the Akt/mTOR signaling pathway (Johnson et al., 2009). Curcumin as an antitumoral agent is currently under phase II clinical development for prevention of colorectal cancer. Pharmacokinetic studies show that the absorbed curcumin conjugates with glucuronic acid and sulfate, and metabolized to glucuronic acid and sulfate conjugates in the intestine and liver (Ireson et al., 2002). Excretion of curcumin glucuronides from intestinal cells occurs predominantly at the apex and to a lesser extent at the basolateral side, thus limiting its absorption (Usta et al., 2007). In cancer patients, the serum concentration peaks at 1–2 h after intake and does not exceed 0.60 μg/mL (0.16 μmol/L) even at a dose of 8 g curcumin per day for 3 months (Cheng et al., 2001). In a dose-escalation study, curcumin was not detected in the serum for up to 4 h after administration of single doses of 0.5–8.0 g curcuminoids, and only low concentrations between 0.03 and 0.06 μg/mL were detected after single oral dose of 10 or 12 g (Lao et al., 2006). The low bioavailability of curcumin is attributed to its limited absorption, efficient re-secretion from intestinal cells and rapid intestinal and hepatic conversion to its metabolites dihydro-, tetrahydro-, and hexahydrocurcumin and their respective conjugates with glucuronic acid and sulfate, which considerable hampers its therapeutic efficacy and clinical application (Schiborr et al., 2010). Recently, the nanoparticle of curcumin was developed, and the bioavailability of the compound was greatly improved (Zhang et al., 2014).

*Tripterygium wilfordii Hook. f*., distributed in Asia, is another herb used in TCM to treat autoimmune and inflammatory diseases such as rheumatoid arthritis and tuberculosis. Celastrol (Figure 2), a triterpenoid isolated from the plant has attracted great attention due to its potent anticancer effects and its diverse molecular targets involved in tumorigenesis (Yang H. et al., 2006; Sethi et al., 2007; Huang et al., 2008; Peng et al., 2010; Kannaiyan et al., 2011; Lee et al., 2012; Rajendran et al., 2012). The anticancer mechanisms of the compound include inducing apoptosis in tumor cells, affecting angiogenesis, regulating the related proteins of tumor and so on. Recent studies have shown that Reactive oxygen species (ROS)/c-Jun NH2-terminal kinase (JNK) signal pathway plays a critical role in celastrol induced cell apoptosis; treatment of cancer cells with celastrol activates caspase-3, -8, -9, DR5, and cleavage of PARP, Bid, upregulates the expression of LC3B-II. The augmentation of JNK phosphorylation and ROS generation is another important event in celastrol induced cell apoptosis (Li H. Y. et al., 2015). In addition, celastrol is able to suppress the expression of vascular endothelial growth factor receptor (VEGFR), and inhibits the growth of human glioma xenografts in nude mice (Huang et al., 2008). Additionally, treatment with celastrol resulted in significant inhibition of the tumor growth without host toxicity in nude mice bearing prostate tumors (Yang H. et al., 2006). These studies suggest that celastrol is a promising candidate for development as an anticancer agent. A sensitive and precise LC–MS/MS assay was developed to determine the pharmacokinetic profiles of celastrol in rats (Zhang et al., 2012). The results showed that oral administration of pure celastrol leads only to an bioavailability of 17.06%, whereas after oral administration of TGV tablets, the absolute bioavailability of celastrol in female rats increased up to 94.19%, demonstrating improved absorption properties of celastrol (Zhang et al., 2012).

*Chelidonium majus L*. is used in TCM to treat ulcer and gastrointestinal pain. Chelerythrine (Figure 2) is a benzene alkaloid isolated from the herb. Chelerythrine has been proved to possess potent antitumor effect on various cancers, in particular breast, colon and prostate cancers (Chmura et al., 2000). The mechanism of action of chelerythrine involves several pathways, including cell cycle arrest andinhibition of protein synthesis. Chelerythrine activated JNK/p38 MAPK pathways in a concentration and time-dependent manners in Hela cervical cancer cells (Yu et al., 2000). Treatment of chelerythrine resulted in activation of MEK/ERK1/2 signaling pathway, up-regulation of downstream kinases (p90RSK), and finally induction of apoptosis in human osteosarcoma cells (Yang et al., 2008). Chelerythrine can also induce G1 phase arrest and bimodal cell death in human leukemia HL-60 cells (Vrba et al., 2008). In addition, chelerythrine is a specific inhibitor of protein kinase C (PKC), blocking PKC translocation from cytosol to membrane, contributed to the progression of apoptotic tumor cell death (Siomboing et al., 2001). Recent study also demonstrated that chelerythrine possesses the activity of inhibiting the telomerase activity and promoting cancer cell death via binding with human telomeric DNA to form the four-stranded G-quadruplex (Yang et al., 2011).

Emodin (Figure 2) is an active ingredient derived from the rhizome of *Rheum palmatum L*., which is widely used in TCM as a laxative over thousands years (Ma and Li, 2014; Qu et al., 2015). In the last decades, increased attention is focused on the anticancer activities of emodin since studies have shown that the compound exhibited the effects of antiproliferation and apoptosis-induction in a number of human cancers such as colon, cervical and gastric cancer (Yaoxian et al., 2013; Xie et al., 2014). Emodin inhibited proliferation and induced apoptosis of hepatocellular carcinoma cells both *in vitro* and *in vivo* through MAPK and PI3K/AKT signaling pathways in a dose-dependent manner (Lin et al., 2016). Emodin signifcantly activates the phosphorylation of ERK and p38, which associated with apoptosis of hepatocellular carcinoma (HCC) cells. Moreover, emodin can induce apoptosis of colorectal cancer cells through activating p53/p38/Puma pathway by triggering ROS production (Liu et al., 2015). Pharmacokinetic study revealed that emodin was predominantly found in liver and brain after oral intake of *Polygonum cuspidatum*, which is a widely used in TCM (Lin et al., 2012). After intragastric administration at doses of 20 and 40 mg/kg, emodin rapidly underwent phase II metabolism to form its glucuronide derivative, and the parent form of emodin was almost undetectable *in vivo* (Shia et al., 2010). Glucuronidation metabolism appeared to be one of the main reasons for the very poor oral bioavailability of emodin as found in a cultured Caco-2 cell model (Liu et al., 2012).

*Bolbostemma paniculatum (Maxim.) Franquet* is used in TCM to treat swollen skin, tuberculosis and abscess of the lung. Tubeimoside-1 (Figure 2) as a novel compound with potent anticancer activity is isolated from the plant (Yin et al., 2011; Yu et al., 2001). Tubeimoside-1 inhibited the growth of several cancer cells including gliomas, lung cancer and liver cancer (Zhang et al., 2011; Wang et al., 2011a; Jia et al., 2015). Tubeimoside-1 induced phosphorylation of apoptosis signal-regulating kinase 1 (ASK-1) and its downstream target proteins JNK and p38 in a dose-dependent manner, leading to mitochondrial apoptosis in DU145 human prostate cancer cells (Yang et al., 2016). Activation of MAPK-JNK signaling pathway plays an important role in tubeimoside-1 induced cell cycle arrest in lung cancer cells (Hao et al., 2015). Tubeimoside-1 can also sensitize cell response to cisplatin in cisplatin-resistant human ovarian cancer cells (A2780/DDP) through down-regulation of ERK and up-regulation of p38 (Liu H. Z. et al., 2011). Tubeimoside-1 increased the expression of CHOP and phosphorylated p38, resulting in G_2_/M phase arrest and apoptosis in SKOV-3 human ovarian carcinoma cells (Chen W. J. et al., 2012). In addition, tubeimoside-1 can induce oxidative stress-mediated apoptosis and G_2_/M phase arrest in HepG2 liver cancer cells via NF-κB, JNK, and p53 pathways (Yin et al., 2011). LC/MS analysis was performed to check the pharmacokinetics of tubeimoside-1 after intravenous and oral administration in rats (Liang et al., 2007). Tubeimoside-1 was found with very slow clearance via hepatic tissues. The absolute oral bioavailability of tubeimoside-1 was only 0.23%, suggesting that tubeimoside-1 has poor absorption or undergoes acid-induced degradation.

*Atractylodes lancea rhizome* is recognized to possess the diuretic and stomachic effects in TCM, and used to treat abdominal distention and dismembered sores in China. Two oil products, β-eudesmol (Figure 2) and hinesol (Figure 2) are isolated from the plant. Recent study showed that β-eudesmol is able to activate JNK/MAPK signaling pathway, and induce cell death through mitochondria-mediated intrinsic apoptosis modulated by JNK-dependent downregulation of Bcl-2 in HL60 leukemiacells (Li Y. et al., 2013). β-eudesmol induced the decrease of matrix metalloproteinases (MMP) and the release of cytochrome C from mitochondria in HL60 leukemia cells accompanied with the activation of caspase-9, caspase-3, and cleavage of PARP. β-eudesmol exhibited the inhibitory effect on the growth of various cancer cells including HeLa cervical cancer, SGC-7901 gastric cancer, and liver cancer BEL-7402 cells *in vitro* (Tsuneki et al., 2005). Hinesol, a sesquiterpenoid component isolated from the herb, also induced apoptosis via JNK signaling pathway. Hinesol treatment significantly activated JNK and ERK, but did not alter the activation of p38; thus hinesol may represent a novel anticancer agent in the treatment of leukemia (Masuda et al., 2015).

*Bidens bipinnata L*. has been used in TCM as a basic drug historically in the local area of Guangxi, China, to treat many kinds of diseases such as malaria, diarrhea, dysentery, hepatitis, acute nephritis, hypertension, hyperlipidaemia, and diabetes. Isoquercitrin (Figure 2) is a favonoid compound with anticancer activity isolated from *Bidens bipinnata L* (Yang et al., 2013; Wu et al., 2013a). Isoquercitrin strongly inhibited the phosphorylation of ERK and p38MAPK proteins while promoting the phosphorylation of JNK, thus inducing apoptosis in HepG2 liver cancer cells in a caspase -dependent manner (Huang et al., 2014). Isoquercitrin can also block the liver cancer cells at the G_1_ phase and exhibited inhibitory effect on transplanted tumor growth *in vivo* (Huang et al., 2014).

The roots of *Echinops grijsii*, is believed to possess the effects of antiinflammation, detoxicating, and expelling miasma in TCM, and used to relieve the distention of the breast and stimulating milk secretion (Jin et al., 2008; Zhang et al., 2008). A thiophene derivative, 2-(Pro-1-ynyl)-5- (5,6-dihydroxypenta-1,3-diynyl) thiophene (PYDDT) (Figure 2) is isolated from the herb. PYDDT induces the production of ROS, and the activation of JNK but not p38 and ERK1/2, leading to induction of mitochondrial-mediated apoptosis in human colon cancer SW620 cells (Xu et al., 2015). PYDDT-induced apoptosis was characterized by the cleavage of PARP, activation of caspase 9 and caspase 3, release of cytochrome C from mitochondria, loss of mitochondrial membrane potential, down-regulation of Bcl-2, and mitochondrial translocation of Bax.

*Fagopyrum tataricum (L.) Gaertn (tartary buckwheat)* has been widely used as an important folk medicine in China as a nutritional food. Studies have shown that the herb has multiple benefits including antioxidant, antitumor, antihypotensive, hypoglycemic, and hypolipidaemic effects (Lin et al., 2011; Karki et al., 2013). Tatariside G (Figure 2), a novel phenylpropanoid glycosides compound, was isolated from the roots of *Fagopyrum tataricum (L.) Gaertn*. Recent study revealed that tatariside G notably inhibited cell viability and induced apoptosis in human cervical cancer HeLa cells through both activation of p38/JNK phosphorylation and inhibition of Akt phosphorylation (Li et al., 2014). Tatariside G could elevate the cleaved protein expression of caspase-3 and caspase-9 in a dose-dependent manner, and decreased mitochondrial membranep potential (MMP) in HeLa cells (Li et al., 2014).

## Components isolated from TCMs targeting the phosphoinositide kinase (PI3K)/serine/threonine-specific protein kinase (AKT)

The PI3K signaling pathway contributes to tumor development and progression in many types of human malignancies. It is well acknowledged that activation of AKT, the major downstream effecter of PI3K, is frequently observed in human tumors (Brugge et al., 2007; Yu et al., 2012), and the activation of AKT promotes the development of cancer as well as resistance to chemotherapy and radiation therapy. Additionally, immunohistochemical analysis has shown that AKT activation is a poor prognostic factor in various cancers (LoPiccolo et al., 2007). Therefore, PI3K/AKT signaling pathway is an attractive target for cancer therapy (Hennessy et al., 2005; Crowell et al., 2007). Several components isolated from TCMs were found to induce cell death via inhibiting PI3K/AKT pathway.

**Figure 3:**
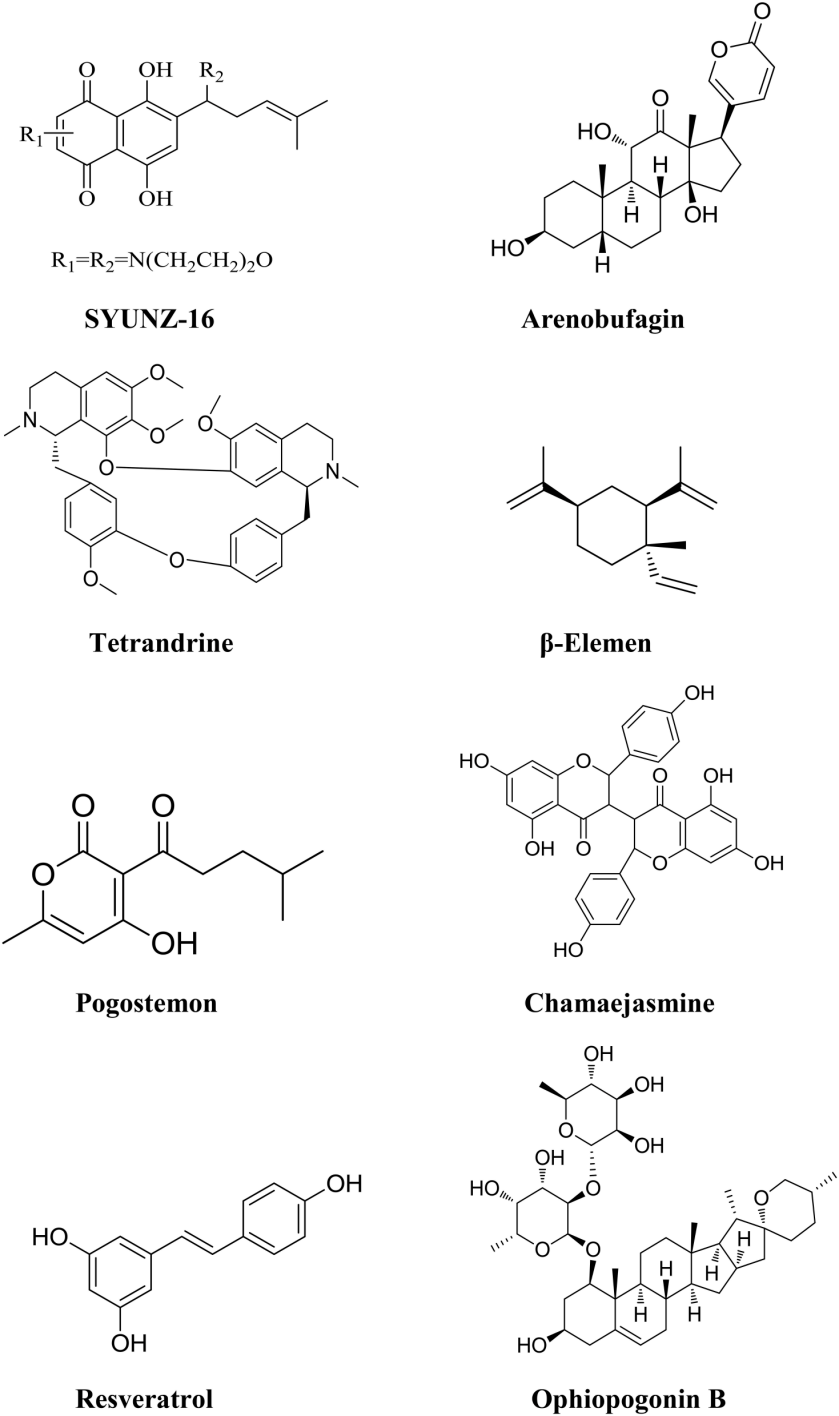
**Chemical structures of components isolated from TCMs with inhibitory effects on PI3K/AKT pathway**.

Alkannin (Figure 3) is the major active ingredient isolated from *Arnebia euchroma roots*, which has long been used as anti-inflammation and anti-tumor herb in Chinese folk medicine (Feng et al., 2003). Studies have shown that alkannin exerted antitumor effects by inhibiting cancer cell proliferation and inducing apoptosis via inhibiting DNA topo-isomerase I/II activity, anti-telomerase activity and anti-angiogenesis (Lu et al., 2002; Yang F. et al., 2006; Lim et al., 2007). SYUNZ-16, a synthesized alkannin derivative, is one of the compounds with potent antitumor activities (Wang et al., 2006; Xie et al., 2006a,b, 2007). SYUNZ-16 displayed potent cytotoxicity in diversified cancer cell lines including nasopharyngeal carcinoma, hepatocellular cancer, leukemia, cervical cancer, gastric cancer and breast cancer. SYUNZ-16 inhibits AKT signaling pathway, and down-regulates the phosphorylation of AKT in a dose and time-dependent manner, subsequently initiating apoptotic events in Hep3B liver cancer and GLC-82 lung cancer cells (Deng et al., 2010). SYUNZ-16 can obviously inhibit the proliferation of these cancer cells via induction of apoptosis with the activation of caspase -3 and PARP cleavage (Deng et al., 2010). SYUNZ-16 can also partially attenuate the phosphorylation levels of forkhead transcription factors (FKHR and FKHRL1), which are important substrates of AKT (Tokunaga et al., 2008). SYUNZ-16 exhibits inhibitory effects on murine S-180 sarcoma allografts and GLC-82 lung cancer xenografts *in vivo* (Deng et al., 2010).

Toad venom (venenum bufonis, also called Chan'su) is derived from the dried skin secretions of giant toads (*Bufo gargarizans Cantor or Bufo melanostictus Suhneider*) and has been widely used alone or in combination with other herbal ingredients for cancer treatment over centuries in China. An injectable formulation of toad venom called cinobufacin (Huchansu) was developed to treat various solid tumors in China in 1990's. Clinical studies have shown that cinobufacin significantly increased the antitumor efficacy of docetaxol or cisplatin in the combination therapy. Decreased toxicity and improved life quality were also observed in the clinical trial with cancer patients (Gong et al., 2010). Arenobufagin (Figure 3), one of the components of toad venom, was reported to have a broad spectrum of antitumor activity in cancer cells, including MCF-7, MCF-7/ADR, HepG2, and some other carcinoma cell lines (Zhang et al., 1992; Masuda et al., 1995; Yu et al., 2010). PI3K/AKT signaling pathway plays a central role in arenobufagin-mediated cell death. Arenobufagin can inhibit AKT with involvement of Phosphatase and tensin homolog (PTEN) activation as well as PDK1 and PI3K inhibition, and induces apoptosis in HCC cells. Arenobufagin treatment leads to marked decrease in the expression of mTOR. Inhibition of PI3K/AKT/mTOR pathway can promote the development of both autophagy and apoptosis induced by arenobufagin (Brown et al., 2003; Teachey et al., 2006; Chen G. et al., 2012). Arenobufagin induces mitochondria-mediated apoptosis in HepG2 and HepG2/ADM cells, accompanied with a decrease of mitochondrial potential, and an increase of Bax/Bcl-2 expression ratio. Activation of caspase-3 and caspase-9 as well as cleavage of PARP was also found in arenobufagin induced cell apoptosis (Zhang D. M. et al., 2013). In addition, arenobufagin is able to block VEGF-mediated angiogenesis (Li M. et al., 2013). Therefore, arenobufagin as the main active ingredients of toad venom has the potential to be developed as a novel anticancer agent (Liu et al., 2009; Hu et al., 2011). The pharmacokinetic characteristics of arenobufagin has been studied in six Sprague-Dawley rats by ultra-fast liquid chromatography–tandem mass spectrometry (Li G. et al., 2013). Arenobufagin can be detected in plasma within 5 min to a peak concentration of 1.98 ng /mL after intraperitoneal administration 4.0 mg/kg, which indicates that arenobufagin can be absorbed quickly (Li G. et al., 2013).

*Radix Stephaniae tetrandrae* is used to treat the syndrome of dampness-heat related diseases in TCM over thousands of years and it is widely used to treat cystitis, prostatitis, urethritis, pyelonephritis, vaginitis as well as rheumatism in China. A bisbenzylisoquinoline alkaloid, tetrandrine (Figure 3) is isolated from the roots of *Radix Stephaniae tetrandrae* (Bosch et al., 2004; Liu et al., 2006). Studies have shown that tetrandrine is able to inhibit cell proliferation and induces apoptosis of cancer cells (Yan et al., 2006; Chen et al., 2008). ERK and PI3K-AKT signaling pathways play a critical role in tetrandrine induced cell apoptosis (Lin et al., 2008). Treatment of cancer cells with tetrandrine leads to the suppression of AKT activation, which in turn regulated the function of Bcl-2 family proteins and activated caspase cascades (Liu C. Y. et al., 2011). Tetrandrine also has the potential of immunomodulation and anti-inflammatory activity, which plays a positive role in HCC therapy (Shen et al., 2001). Based on a long history of clinical application in TCM, tetrandrine is considered to be a safe agent, and may be an attractive candidate compound for liver cancer therapy. The Pharmacokinetic properties of tetrandrine were studied with a simple HPLC method in rabbits. The concentration-time data of tetrandrine fitted the classical two-compartment model, whether the drug was administered intravenously or orally (Jiang et al., 2011). The ratio of tetrandrine AUC (10 mg/kg by gavage) to AUC (5 mg/kg by intravenous injection) was about 30% and ratio of their Cmax was less than 20%, suggesting that tetrandrine is less absorbed from digestive tract or has a strong first pass effect as the gavage dose is double that of intravenous dose (Jiang et al., 2011).

*Rhizoma zedoariae* possesses the effects of subsiding swelling, relieving pain in TCM and used to tread rheumatalgia, headache and chestpain. Elemene (1-methyl-1- vin- yl-2,4- diiso- propenyl-cyclohex-ane, Figure 3), a noval lipid-soluble component, is extracted from the rhizoma of zedoariae (Li et al., 2009). β-Elemene, the most active component of elemene, has been shown to be effective against various cancers *in vitro* and *in vivo*, such as lung cancer, colorectal cancer and glioblastoma (Wang et al., 2005; Yao et al., 2008; Li et al., 2009; Xie et al., 2009). Recently, β-Elemene has been approved by the State Food and Drug Administration of China for the treatment of some solid tumors (Tan et al., 2000). β-Elemene induces apoptosis and autophagy through inhibition of the PI3K/Akt/ mTOR/p70S6K1 signaling pathway in human gastric cancer cells (Liu J. et al., 2011). Following treatment with β-Elemene, the level of phospho-AKT was obviously downregulated, leading to the down-regulation of downstream phosphor-mTOR as well as phospho- p70S6K1. The cleavage of PARP and conversion of LC3 I to LC3 II is consistent with the change of PI3K/Akt/mTOR/p70S6K1 activity. β-Elemen can induce G2/M phase arrest and apoptotic cell death in non-small lung cancer cells with activation of caspases -9, -3, and 7 (Wang et al., 2005). β-Elemen promotes apoptosis through inhibiting the expression of Bcl-2 and survivin in MCF-7 human breast cancer cells (Hu et al., 2004). In addition, β-Elemen can suppress the expressions of VEGF, basic fibroblast growth factor (bFGF), and epidermal growth factor (EGF), and exhibit anti-cancer ability on laryngeal cancer cells both *in vitro* and *in vivo* (Tao et al., 2005, 2006). β-Elemenal was a primary metabolite in bile of rat after β-Elemen intravenous administration. A sensitive gas chromatographic–mass spectrometric assay was developed to determine the level of β-Elemen and β-Elemenal in human plasma (Chen et al., 2009). The peak plasma concentration (Cmax) and area under curve (AUC) of β-Elemen were prone to increase in proportion to the dose, but there were no significant differences among Cl values in the range of dosages. Moreover, no β-Elemenal was detected in plasma, and there was no other obvious homologous fragment elsewhere, which indicated that β-Elemen may be mainly decomposed into some small hydrophilic metabolites. Further investigations are needed to determine the biological process of β-Elemen *in vivo* (Chen et al., 2009).

*Pogostemon Cablin (Blanco) Benth*, commonly known as “Guang-huoxiang” in China, is a TCM herb widely used to treat gastrointestinal diseases in many Asian countries (Chen et al., 2015). Pogostone (Figure 3) is one of the major constituent of Pogostemon cablin, and possesses various bioactivities, such as anti-fungal (Li et al., 2012), anti-bacterial (Peng et al., 2014), pesticidal (Huang et al., 2013), and anti-inflammatory activities (Su et al., 2015). Recent studies have revealed that pogostone exhibited potent anti-proliferative activities against multiple human cancer cell lines, especially on human colorectal cancer cells HCT116 (IC50: 18.7 ± 1.93 μg/mL; Cao et al., 2016). Pogostone significantly inhibited AKT and mTOR phosphorylation in a dose-dependent manner, which contributed to the initiation of autophagy and apoptosis in HCT116 cells (Cao et al., 2016). After treatment of pogostone, a dose-dependent increase in the levels of LC3 -II, cleaved caspase-3 and caspase-7, and a significant decrease in pro-caspase-3 levels were observed in HCT116 cells. Pogostone also inhibited the growth of HCT116 tumor, and reduced the tumor volume significantly with well tolerated by the host in *vivo*. Pogostone may be developed as a promising drug in the treatment of human colorectal cancer. The preclinical pharmacokinetic investigation of pogostone has been performed in rats after intravenous and oral administration (Chen H. et al., 2013). The results showed that the blood concentration of pogostone appeared to increase nonproportionally between 5 and 20 mg/kg under the intravenous route (Chen H. et al., 2013).

*Stellera chamaejasme L*. is used to treat skin ulcer and abdominal distension in TCM. Chamaejasmine (Figure 3), a flavone compound isolated from *Stellera chamaejasme L*, displays potent cytotoxicity in multiple cancer cell lines, including human lung cancer A549 cells, and human breast cancer MDA-MB-231 cells (Yu et al., 2011; Zhang T. et al., 2013; Yang et al., 2015). Recent study showed that chamaejasmine could induce apoptosis in HeLa cervical cancer cells, mediated through PI3K/Akt signaling cascades (Qiang and Li, 2017). Treatment of chamaejasmine inactivates AKT to trigger apoptosis in human hep-2 larynx carcinoma cells (Wang et al., 2011b).

*Polygonum cuspidatum* is believed to possess the effects of dissipating blood stasis and pain relief. Resveratrol 3,4,5-trihydroxystilbene, (Figure 3), a polyphenol compound, is isolated and extracted from *P. cuspidatum* with broad bioactivity including anti-bacterial, anti-inflammatory, anticancer, anti-hyperlipidemia anti-lipid peroxidation and pro-apoptotic effects (Piotrowska et al., 2012; Kucinska et al., 2014). Resveratrol inhibited PI3K and Akt phosphorylations, and subsequently triggered the dephosphorylation of glycogen synthase kinase 3 beta (GSK3β), which resulted in cyclin D1 degradation and eventually cell cycle arrest and apoptosis in MGC803 human gastric cancer cells (Jing et al., 2016). Furthermore, resveratrol can inhibit the invasion and metastasis of colorectal cancer cells through metastasis associated lung adenocarcinoma transcript 1 (MALAT1) mediated Wnt/β-catenin signal pathway (Ji et al., 2013). However, the water solubility of resveratrol was very poor with approximately 0.03 mg/mL (Vian et al., 2005). *In vivo* pharmacokinetic study confirmed that the oral bioavailability of resveratrol approaches zero, although administrated with relatively high concentrations of the compounds (Wenzel and Somoza, 2005). Therefore, it is important to enhance the bioavailability of resveratrol, which is considered as the main challenge in successfully applying resveratrol in clinical and health-promoting interventions (Chang et al., 2016).

Ophiopogonin B (OP-B, Figure 3) is a bioactive component of *Radix Ophiopogon Japonicus*, which is often used in TCM to treat pulmonary disease (Wang Y. H. et al., 2011). OP-B can significantly decrease cell viability in a panel of NSCLC cell lines. OP-B inhibited the PI3K/Akt/mTOR/p70S6K signaling pathway, suppressed p-AKT at both Ser308 and Thr473 and induced autophagy in NCI-H157 and H460 human lung cancer cells (Chen M. et al., 2013). As a prospective inhibitor of AKT/mTOR, OP-B can also exhibit autophagy-dependent antitumor effects via repression AKT/mTOR signaling pathway in human cervical cancer HeLa cells (Xu et al., 2013). OP-B can induce autophagy and apoptosis in A549 human lung cancer cells both *in vitro* and *in vivo* (Chen et al., 2016). Moreover, OP-B significantly decreases cell proliferation and induces apoptosis in SGC-7901 human gastric cancer cells via triggering the JNK1/2 and ERK1/2 signaling pathways (Zhang et al., 2016).

## Components isolated from TCMs that target epidermal growth factor receptor (EGFR)

EGFR (also known as erbB1 or HER1) belongs to the family of tyrosine kinase receptors that include erbB2 (Neu, HER2), erbB3 (HER3), and erbB4 (HER4). EGFR once combined with EGF can promote the related genes in the cell nucleus, leading to cell proliferation. EGFR is commonly highly expressed in a variety of malignant tumors (Nakao and Fusetani, 2007), and the abnormal activation of EGFR is closely correlated with tumor cell biology, acting as an indicator of poor prognosis for the patients with cancer.

**Figure 4:**
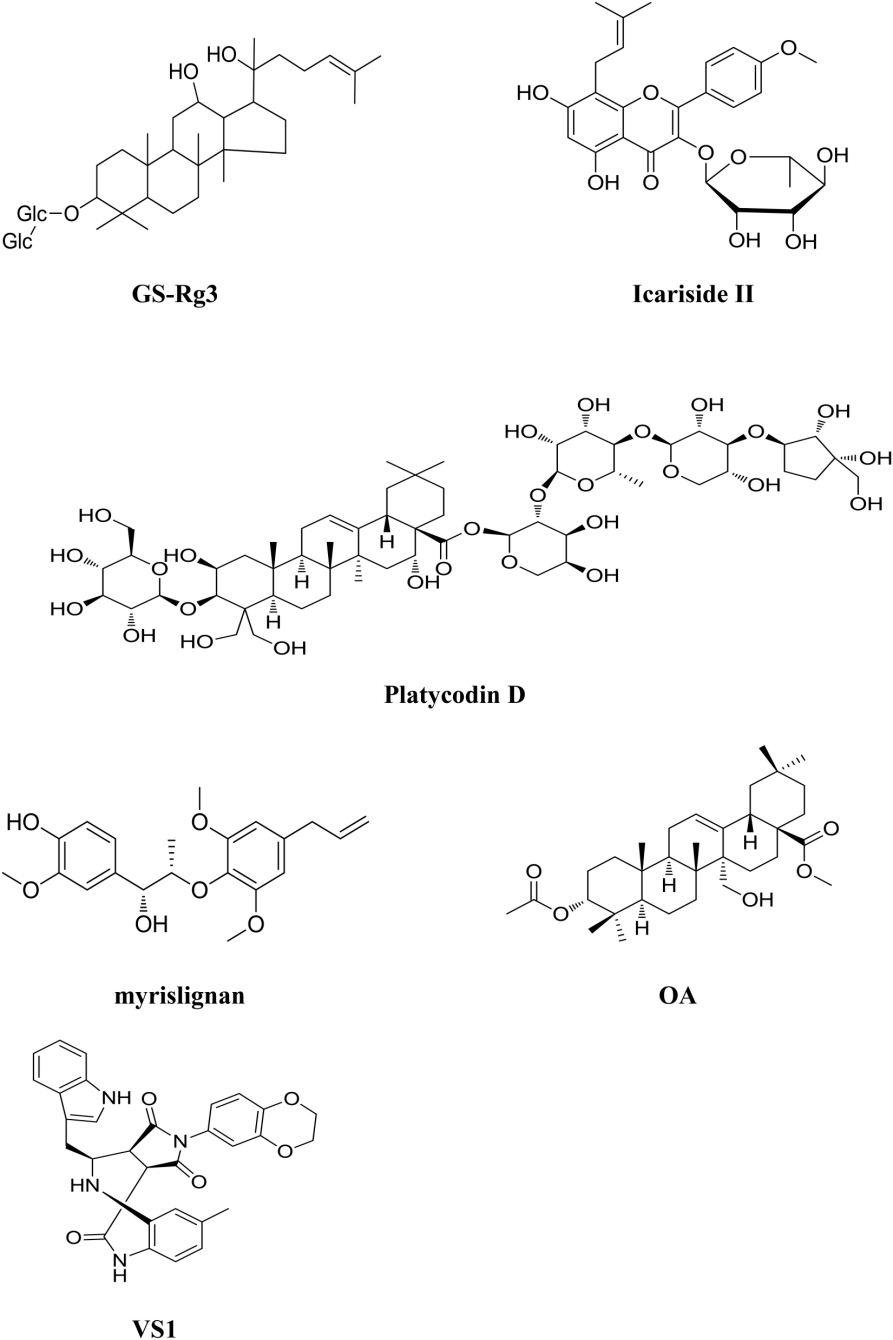
**Chemical structures of components isolated from TCM that target Epidermal Growth Factor Receptor (EGFR)**.

The root of *Panax ginseng C. A. Mey (Gingsheng)* is believed to possess the activity of nourishing vitality and is widely used in China for patients with poor health condition. Ginsenoside Rg3 (GS-Rg3, Figure 4) is one of the active ingredients in Ginsheng with significant antitumor activity. It is also the main component of Shenyi capsule, the first drug used for controlling the metastasis and recurrence of cancer patients in China. GS-Rg3 shows antitumor effects in a variety of cancers such as gastric, lung, colon, breast, and liver cancers etc. (Lu et al., 2008; He et al., 2011). GS-Rg3 displays various anticancer activities including inhibiting tumor growth, invasion and metastasis, and suppressing angiogenesis in tumor tissues and improving immunity. Synergistic anticancer effects are found when it combined with chemotherapeutic agents (Keum et al., 2003). GS-Rg3 inhibited epithelial-mesenchymal transition (EMT) and invasion of lung cancer by down-regulating fucosyltransferase 4 (FUT4) mediated EGFR inactivation and blocking MAPK and NF-κB signal pathways (Shan et al., 2015; Tian et al., 2016). GS-Rg3 reduced the expressions of EGFR and pEGFR in MCF-7 breast cancer cells in a dose-dependent manner, suggesting that GS-Rg3 inhibits the tumor growth by targeting EGFR and its down- stream signal transduction pathways (Wang et al., 2008). GS-Rg3 is also an inhibitor of VEGF and bFGF; significantly decreasing the expression of these angiogenesis factors in human A549 lung cancer and human umbilical vein endothelial cells (HUVEC) (Chen et al., 2005). *In vitro* as well as *in vivo* study have been carried out to determine the blood level of GS-Rg3 in rat plasma and its major metabolites using an HPLC/Q/TOF analytical approach. GS-Rg3 has an average half-life of 18.5 min after intravenous administration dosed at 5 mg/kg, whereas it was not detected in rat plasma after oral administration at 100 mg/kg (Qian et al., 2005). GS-Rg3 was metabolized to ginsenoside Rh2 and protopanaxadiols (PPD) when anaerobically incubated with human fecal microflora, and the deglycosylated metabolites display activities comparable to or higher than that of GS-Rg3 (Xie et al., 2005). However, GS-Rg3 has poor solubility and oral bioavailability, which limits its clinical application. Recently, a derivative of the compound, 20(S)-ginsenoside Rg3, was designed and developed as a new drug. Pharmacokinetics has been studied in healthy volunteers in China (Zhao et al., 2016). 20(S)-ginsenoside Rg3 was generally well tolerated, and exhibited a pharmacokinetic profile suitable for once-every-2-days dosing regimen (Zhao et al., 2016).

*Epimedium koreanum Nakai* is believed to possess the effects of nourishing Yin and strengthening Yang, promoting blood circulation. Icariside II (Figure 4), a flavonoidglycoside compound, is isolated from the stems and leaves of epimedium koreanum Nakai (Aramwit and Wirotsaengthong, 2012). Studies have shown that icariside II exhibited potent cytotoxicity against a broad spectrum of human cancer cells through various pathways (Kang et al., 2012; Wu et al., 2012). Icariside II displays significant antitumor activity against A431 human epidermoid carcinoma cells *in vitro* and in mice bearing osteosarcoma sarcoma-180 *in vivo* by suppressing the phosphorylation of EGFR, down-regulating EGFR downstream signal PI3K/AKT and Raf/MEK/ERK as well as mTOR pathways in these cancer cells (Wu et al., 2013b; Geng et al., 2014). Icariside II metabolites in rats were analyzed using an ultra-performance liquid chromategraphy/quadrupole- time-of-flight mass spectrometry method. The results showed that the metabolized mainly via desugarisation, dehydrogenation, hydrogenation, hydroxylation, demethylation, glucuronidation, dehydration, and glycosylation pathways *in vivo*. Specific hydrolysis of 7-O glucoside in the gut lumen and glucuronic acid conjugation in the liver was considered as the main physiologic processes of icariside II (Sun et al., 2014).

The root of *Platycodon grandiflorum (Companulaceae)* has been extensively used to treat several types of chronic inflammatory diseases in TCM (Shin et al., 2002). Platycodin D (PD, Figure 4), one of the major saponin components contained in the herb is reported to display antitumor effect on several cancer cell lines (Chun et al., 2013). EGFR/PI3K/AKT pathway plays a critical role in PD induced cell apoptosis and PD downregulates the expression of EGFR in MDA-MB-231 breast cancer cells subsequently leading to the inhibition of the PI3K/AKT and MAPK pathways (Chun and Kim, 2013). Additionally, PD could induce apoptosis and trigger ERK- and JNK-mediated autophagy in human hepatocellular carcinoma BEL-7402 cells (Li T. et al., 2015).

The seed of *Myristica fragrans Houtt (Nutmeg)* is used to treat diarrhea and ep-igastric pain in TCM. Recent study showed that one of the components myrislignan (Figure 4) isolated from nutmeg, displayed potent anticancer activity against A549 lung cancer cells both *in vitro* and *in vivo* (Lu et al., 2016). The effects of myrislignan on apoptosis and cell proliferation are mediated by activation of MAPK and inhibition of EGFR signal pathway.

*Peganum harmala L*. is used to treat cancer in TCMs and Uygur medicine. A novel compound called 3α-acetoxy-27-hydroxyolean-12-en-28-oic acid methyl ester (OA, Figure 4) was isolated from the herb (Wang et al., 2016). OA possesses potent anticancer activity against NSCLC via inhibiting the activation of EGFR and its downstream signals. Guo et al. developed a model to identify the ERBB3 inhibitors from natural products and TCMs. Several compounds with anticancer activity were identified; among them, VS1 (Figure 4) is the most promising component with IC_50_ value of 269 μM against A549 lung cancer cells (Guo et al., 2016).

## Conclusions

The discussed components here are isolated from TCMs and their modes of actions are summarized in Table 1. However, it should be kept in mind that numerous components from TCMs display antitumor activity via multiple targets. Most components discussed above show pro-apoptotic activity mediated by activation of caspases and downregulation of mitochondrial antiapoptotic proteins.

**Table 1:** **A summary of Traditional Chinese Medicines as kinase inhibitors**.

**Name of Anti–tumor TCMs**	**Sources**	**Classification**	**Kinase inhibitors and cell lines**	**References**
Curcuma	*Curcuma longa L*.	Polyphenols	JNK/p38 MAPK/ERK HCT-116, THP-1, CNE1, CNE2, and HepG2 cells	Collett and Campbell, 2004; Chintana et al., 2011; Yang et al., 2012; Wang et al., 2013
Celastrol	*Tripterygium wilfordii Hook. f*.	Triterpene	JNK HOS, MG-63, U-2OS, and Saos-2 cells	Li H. Y. et al., 2015
Chelerythrine	*Chelidonium majus L*.	Benzophen anthridine alkaloid	JNK/p38 MAPK/ERK Hela, HOS, and U-2OS cells	Yu et al., 2000; Yang et al., 2008
Emodin	*Rheum palmatum L*.	Anthraquinone	ERK/p38 MAPK SMMC-7721, SW480, and SW620 cells	Liu et al., 2015; Lin et al., 2016
Tubeimoside-1	*Bolbostemma paniculatum Franquet*	Triterpenoid saponin	JNK/p38 MAPK/ERK DU145, A549, PC9 A2780/DDP, SKOV-3. and HepG2 cells	Liu H. Z. et al., 2011; Yin et al., 2011; Chen W. J. et al., 2012; Hao et al., 2015; Yang et al., 2016
β-eudesmol	*Atractylodes lancea rhizome*	Sesquiterpenol	JNK HL60 cell	Li Y. et al., 2013
Hinesol	*Atractylodes lancea rhizome*	Sesquiterpenol	JNK/ERK HL60 cell	Masuda et al., 2015
Isoquercitrin	*Bidens bipinnata L*.	Favonoid	JNK/p38 MAPK/ERK HepG2 cell	Huang et al., 2014
PYDDT	*Echinops grijsii*	Alkynol group- substituted thiophene	JNK SW620 cell	Xu et al., 2015
Tatariside G	*Fagopyrum tataricum (L.) Gaertn*	Phenylpropan- oid glycosides	JNK/p38 MAPK HeLa cell	Li et al., 2014
SYUNZ-16	*Arnebia euchroma roots*	b,b-dimethylacrylalkannin	AKT Hep3B and GLC-82 cells	Deng et al., 2010
Arenobufagin	*Bufo gargarizans Cantor or Bufo melanostictus Suhneider*	C24 steroids	PI3K/Akt/mTOR HepG2 and HepG2/ ADM cells	Zhang D. M. et al., 2013
Tetrandrine	*Radix Stephaniae tetrandrae*	Bisbenzylisoquinoline alkaloid	ERK and PI3K/AKT HT-29, Huh7, HepG2 and BEL7402 cells	Chen et al., 2008; Liu C. Y. et al., 2011
β-Elemene	*Rhizomazedo- ariae*	Terpene	PI3K/Akt/mTOR MGC803 and SGC7- 901cells	Liu C. Y. et al., 2011; Liu J. et al., 2011
Pogostone	*Pogostemon Cablin (Blanco) Benth*	Ketone	PI3K/Akt/mTOR HCT116 cell	Cao et al., 2016
Chamaejasmine	*Stellera chamaejasme L*.	Flavonoid	PI3K/Akt HeLa and HEp-2 cells.	Wang et al., 2011b; Qiang and Li, 2017
Resveratrol	*Polygonum cuspidatum*	Polyphenol	PI3K/Akt MGC803 cell	Jing et al., 2016
Ophiopogonin-B	*Radix Ophiopogon Japonicus*	Saponin	PI3K/Akt/mTOR NCI-H157, H460 and HeLa cells	Chen M. et al., 2013; Xu et al., 2013
Ginsenoside Rg3	*Ginsheng*	Saponin	EGFR and VEGF A549, H1299, H358, MCF-7 and HUVEC304 cells	Chen et al., 2005; Wang et al., 2008; Tian et al., 2016
Icariside II	*Yin Yanghuo Horny Goat Weed*	Flavonoid	EGFR A431 cell	Wu et al., 2013b; Geng et al., 2014
Platycodin D	*Platycodon grandiflorum*	Saponin	EGFR MDA-MB-231 cell	Chun and Kim, 2013
Myrislignan	*Myristica fragrans Houtt*	Lignans	EGFR A549 cell	Lu et al., 2016
OA	*Peganum harmala L*.	Triterpene	EGFR A549 cell	Wang et al., 2016
VS1		Heterocycle	ERBB3 A549 cell	Guo et al., 2016

In recent years, TCM has gained increasing acceptance and attention worldwide, and is recognized as a rich source for drug discovery and development. TCM is widely used in China to reduce the side effects of chemotherapeutic drugs and improve the outcome of conventional treatment. It should emphasize that in some cases, the mixed extracts of TCM display more potent antitumor effects than the single individual component and exhibited synergistic effects with most TCM preparation. Our recent study revealed that the crude extract of clove bud can induce cell death via apoptotic pathway and inhibit the growth of cancer cells both *in vitro* and *in vivo*. However, the isolated bioactive component, Oleanolic acid, displays much weaker antitumor activity compared to the crude extract in the nude mouse models of xenografted human tumors, suggesting synergistic antitumor effects exist among the components in the extract of clove bud (Liu et al., 2014).

Although, chemotherapy is acknowledged as one of the most effective therapeutic methods for cancers in late stage, it faces serious side effects and drug resistance. TCM provide a novel strategy for cancer therapy. However, the underlying mechanisms of most TCM have not been elucidated yet. On the other hands, numerous components isolated from TCMs possess poor bioavailability; novel approaches, including chemical modification, nanotechnology etc. should be employed to improve their efficacy *in vivo*. It is likely that the efficacy of TCM in cancer treatment may lead to novel strategies in fight against various cancers.

## Author contributions

ML, GZ are Equal contributors and co-first authors. ML, GZ, YZ, and XFL consulted literature about Targeting protein kinases with components from Traditional Chinese Medicine. ML, GZ wrote the review. XKL, SC edited and revised the manuscript critically for important intellectual content.

### Conflict of interest statement

The authors declare that the research was conducted in the absence of any commercial or financial relationships that could be construed as a potential conflict of interest.
